# An ontology-based approach for developing a harmonised data-validation tool for European cancer registration

**DOI:** 10.1186/s13326-020-00233-x

**Published:** 2021-01-06

**Authors:** Nicholas Charles Nicholson, Francesco Giusti, Manola Bettio, Raquel Negrao Carvalho, Nadya Dimitrova, Tadeusz Dyba, Manuela Flego, Luciana Neamtiu, Giorgia Randi, Carmen Martos

**Affiliations:** European Commission, Joint Research Centre, Via E. Fermi 2749, I-21027 Ispra, VA Italy

**Keywords:** Cancer registry, Ontology, Data validation, Data federation, Semantic web, Data harmonisation

## Abstract

**Background:**

Population-based cancer registries constitute an important information source in cancer epidemiology. Studies collating and comparing data across regional and national boundaries have proved important for deploying and evaluating effective cancer-control strategies. A critical aspect in correctly comparing cancer indicators across regional and national boundaries lies in ensuring a good and harmonised level of data quality, which is a primary motivator for a centralised collection of pseudonymised data. The recent introduction of the European Union’s general data-protection regulation (GDPR) imposes stricter conditions on the collection, processing, and sharing of personal data. It also considers pseudonymised data as personal data. The new regulation motivates the need to find solutions that allow a continuation of the smooth processes leading to harmonised European cancer-registry data. One element in this regard would be the availability of a data-validation software tool based on a formalised depiction of the harmonised data-validation rules, allowing an eventual devolution of the data-validation process to the local level.

**Results:**

A semantic data model was derived from the data-validation rules for harmonising cancer-data variables at European level. The data model was encapsulated in an ontology developed using the Web-Ontology Language (OWL) with the data-model entities forming the main OWL classes. The data-validation rules were added as axioms in the ontology. The reasoning function of the resulting ontology demonstrated its ability to trap registry-coding errors and in some instances to be able to correct errors.

**Conclusions:**

Describing the European cancer-registry core data set in terms of an OWL ontology affords a tool based on a formalised set of axioms for validating a cancer-registry’s data set according to harmonised, supra-national rules. The fact that the data checks are inherently linked to the data model would lead to less maintenance overheads and also allow automatic versioning synchronisation, important for distributed data-quality checking processes.

## Background

### Cancer registries

A cancer registry (CR) is defined as an organisation for the collection, storage, analysis and interpretation of data on persons with cancer [1]. A population-based CR has to ensure registration of all cases of cancer within a population of well-defined composition and size to ensure completeness and accuracy of cancer indicators.

Population-based CRs form a critical element in the field of cancer epidemiology, especially in terms of monitoring cancer burden but also for identifying or following up cohort populations for studies on cancer aetiology. Inter-comparison of key cancer indicators such as incidence, mortality, survival and prevalence across different populations is important for ascertaining good practices and for deploying effective control strategies. Europe has a strong history in cancer registration and provides a rich set of research data given the wide variation of life-styles and national/regional health policies across the continent.

Owing to the monitoring capability of population-based registries, the European Commission has been proactively supporting cancer registration in response to calls from the European Parliament and the European Council to address the rising cancer burden [2–4]. One of these initiatives is the harmonisation of a core set of cancer-registration data from which the key indicators for monitoring the burden of cancer can be derived. The European Commission, via its Directorate-General Joint Research Centre (JRC), works in close collaboration with the European Network of Cancer Registries (ENCR) and other stakeholders, such as the International Agency for Research on Cancer (IARC) and the EUROCARE^1^ project to ensure accurate sets of cancer indicators that can be compared across national boundaries.

The organisation of cancer registration in Europe is complex. Some countries have a single national CR whereas others have a looser structure of regional CRs that may or may not cover the entire country. The situation is compounded by the dynamics of possible mergers of registries as regional boundaries change or by the transition towards a national registry from a former regionally based model. Different health-care infrastructures also result in different governance, data-collection, and financing modalities for the CRs. One of the key challenges concerns data harmonisation, which is a critical component for ensuring unbiased comparison of indicators between and within countries.

### ENCR-JRC core data set and derivation of indicators

The provision of country- and region-comparable cancer indicators across Europe has traditionally followed a centralised process whereby a core set of CR data is collected from the individual CRs on the basis of a pre-defined data protocol and thereafter cleaned according to agreed and harmonised data-quality standards.

The ENCR core data set at the time of writing comprises 55 individual harmonised variables (of which 23 are mandatory). Four of these variables relate to dates (dates of birth, incidence, registration, and last-known vital status), for which the day, month, and year each require a separate variable. Considering dates as a single field, the total number of specific information variables then reduces to 46 (of which 17 are mandatory). Several variables provide information describing the tumour – for example topography (tumour location); morphology (tumour form/structure) and behaviour (whether the tumour is benign/in situ/malignant/uncertain); grade (the degree of the abnormality of the tumour cells), and extent of disease (how confined the tumour is). Other information relates to the basis of diagnosis (how the tumour was diagnosed, such as: clinical investigation, cytology, histology, etc.); and to stage (the state of progression of the tumour at diagnosis). The latter is generally described by the TNM Classification of Malignant Tumours globally recognised standard [5] in which alphanumeric codes are used to describe: the size of the tumour (T), the regional lymph nodes involved (N), and the spread of cancer or distant metastasis (M). The codes attributed to each of the components T, N, and M, together determine the stage group of the tumour, which ranges from zero (in situ) to four (spread to other organs).

Mandatory variables include pseudonymised patient and tumour identifiers, age at diagnosis, incidence date, basis of diagnosis, tumour topography, tumour morphology and behaviour, tumour grading, and duration of survival. After the core data sets have been collected from the CRs, they are validated and processed with population and mortality data to derive the epidemiological indicators relating to cancer incidence, mortality, and survival. The indicators are aggregated over age intervals (expressed as five-year age brackets) for each type of cancer, and are used to monitor the trends and differences across Europe as well as for deriving projections and estimates where data is not directly available. These statistics are made available on the European Cancer Information System (ECIS) website [6].

The centralised data collection and validation process is not straightforward and generally time-consuming. First of all, a data protocol has to be defined followed by a formal data call to the CRs, with the subsequent steps of data-validation and cleaning oftentimes requiring a number of iterations. The process is also quite restrictive on the number of data variables that can be collected due to the minimisation principle whereby data have to be limited to the minimum set necessary for the stated intended purposes.

The time constraints inherent in a centralised data-collection process are additional to those already faced by the individual registries themselves in collecting and validating the data at local level from the primary data sources. These time constraints serve to compromise the timeliness of the data, which is itself important for effective feedback into the policy-cycle loop. The stricter regime for data processing required by the EU’s recent general data-protection regulation is likely to present a further challenge to the ease of transferring data and the consequent timely release of validated, harmonised cancer data. Moreover, duplication of data sets leads to issues of data-integrity and increased data maintenance.

For these reasons, a ramp-up in efficiency could be gained by any solution that adequately circumvents the need for a centralised data-validation process. In such a scenario, data validation would be performed according to a harmonised process at the local cancer-registry level where the legal basis for processing of sensitive information has already been established. The validated data would thereafter be aggregated (in such a manner to prevent identification of individuals) also at the local level. The result of this operation would be an anonymised data set that can be shared freely without the constraints of the GDPR for sensitive data.

### Data validation

One of the main difficulties in moving away from a centralised approach concerns the potential degradation in data quality that is critical for determining accurate and comparable cancer statistics.

Efforts have been made to provide a common data-validation tool and to make the underlying requirements on the quality of CR data as transparent as possible. Important steps in this direction have been the free distribution of the JRC-ENCR data-validation software [7, 8] and the publication of a document describing the data formatting and data-element relationships of the core data set on which the software is based [9].

Whereas the availability of these resources is a beneficial development, they are not sufficient to make the data-validation process entirely independent of the central entity. The standards and data relationships are open to interpretation since they are not specifically described in a rigorous, formal manner. In particular, a change in the data-relationship description requires a separate change to the data-validation software giving rise to eventual synchronisation and versioning-control issues.

Moreover, formal data-description techniques and machine-readability are becoming increasingly important for rapidly evolving data-mining techniques and for artificial-intelligence based tools. Secondary data usage often requires the linkage of different types of data sets and relies to an ever greater extent on semantic data models. To address these needs, much progress has been made in the development of semantic web technologies and the underlying tools are becoming mature enough to use with advantage.

Previous studies have pointed to the usefulness of ontologies in data validation and cleaning from the perspective of trapping generic-type errors (such as typographical errors or inconsistent naming conventions) [10] or automated ontology maintenance purposes based on learning from existing stable data sets [11]. Ontologies are an attractive proposition for converging the description of the data model and the associated rule base into a single application. The advantage would be that the data-validation rule base is always in synchronisation with the data model thereby easing maintenance overheads.

Ontologies developed in OWL derive many advantages afforded by the semantic web stack. The purpose of OWL is to represent complex knowledge of entities in a domain via a computational, logic-based language such that the knowledge encapsulated can be verified for consistency or used as a basis for inferences on that knowledge [12]. Moreover, OWL has been used to good effect in providing richer querying capabilities to existing cancer-data resources using semantic information [13, 14]. For example, Esteban-Gil et al. [13] have shown the benefits of a semantically enabled CR for extracting groups of patients based on semantic profiles for analysing disease courses over time. OWL ontologies have also been used in decision aids and training tools for cancer diagnosis and treatment [15].

## Implementation

Using OWL for a data-validation tool is a slight departure from OWL’s primary aims. Rather than using the ontology to validate the consistency of the knowledge base or to make further inferences from the axioms specified in the knowledge base, the task of a data-validation tool is primarily to verify the adherence of data elements to a set of prerequisite and well-defined rules. Data validation lends itself more to terminological-component (TBox) reasoning in a closed-world scenario.

Given its need to gather and relate information from distributed sources on the web, an important design decision of OWL is its inherent open-world assumption (OWA). In the OWA, a statement is considered to be true unless it is explicitly stated to be false, with the consequence that not everything can be known a priori about a particular entity – there may always be new knowledge that extends the information about it. In contrast, the closed-world assumption (CWA) considers a statement to be false unless it has been explicitly stated as true. This topic has been addressed by others who have proposed a number of practical solutions for dealing with predominantly closed-world scenarios [16, 17], essentially by developing semantics of extended DL knowledge bases using the notion of integrity constraints. Implementation of this extension was however under further investigation at the time. Tao et al. [16] were using SPARQL query answering while the intention of Motik et al. [17] was to implement their approach in the KAON2 DL reasoner.

The main advantage for using OWL lies in its flexibility and expressiveness for creating web ontologies, as well as its basis on the Resource Description Framework (RDF) for linking structures and making them directly accessible on the Web via unique Uniform Resource Identifiers (URIs). Other advantages include the wide availability of reasoning tools for OWL ontologies, important for making inferences from quite a complex set of inter-dependent rules, as well as its ability to import separate ontologies. Cancer registration draws on a number of coding standards and terminologies (including, ICD-10 [18], ICD-O-3 [19], TNM Classification of Malignant Tumours [5], SNOMED CT [20]), and where these exist as separate ontologies OWL is able to import them without having to redefine all the associated entities. The difficulties arising from the OWA did not present a major limitation to the use of OWL in this work, as may be seen in the example scenarios given in the Results section.

Two existing ontologies were considered as a potential basis for the data-validation tool. Both ontologies are highly pertinent to the field of cancer registration but are in preliminary form and undergoing further development. The first was developed as a model for integration of disease classifications in oncology (essentially integrating subsets of ICD-10 and ICD-O-3 terminologies) [21]. The second was developed for the analysis and visualisation of disease courses [13]. The purpose of these two ontologies together with the data-validation work described here address three major concerns of population-based CRs. Population-based CRs record all incident cases of cancer in a well-known population. They collect this data from multiple information sources – such as hospital-discharge and clinical records, pathology reports, and death certificates – and oftentimes have to deal with different systems of disease encoding. The aim of the ontology of Jouhet et al. [21] was to facilitate the task of disease identification independent of the coding system used. The subsequent step is to ensure the validity of data using standardised rules, most of which check inter-variable dependencies in the manner described in this paper. Once the data has been validated, it can then be used in data analyses of the type described by Esteban-Gil et al. [13]. These studies generally select cohorts of patients on the basis of specific criteria (e.g. disease courses, patient outcomes, etc.).

These three processes use the data in quite different ways and for quite different purposes. Whereas the goal should be to unite the concepts in a single CR ontology, further study is required to find an optimum solution that addresses each process without adding inconsistencies in the axioms for the other processes or unnecessary overheads to the automatic reasoning functions. For example, the ontology of Jouhet et al. [21] draws on the North American National Cancer Institute thesaurus (NCIt), included in the Open Biological and Biomedical Ontology (OBO) Foundry [22], and the authors note that the ontology suffers a number of flaws, particularly in the logic-based reasoning and should only be used cautiously. The ontology of Esteban-Gil et al. [13] operates on post-validated data and serves as a potential tool for research and knowledge management. It forms part of a larger more complex system for building the queries via SPARQL and imports classes from the Semanticscience Integrated Ontology (SIO) [23] and the Ontology for Biomedical Investigations (OBI) [24].

Although both the ontologies contained a number of common classes (e.g. those deriving from the ICD-O-3 nomenclature), they were structured in a form that would have proved convoluted or restrictive for the data-validation rules. For example, the ICD-O-3 codes in the disease-classification ontology were modelled as individuals of type equating to ICD-10 classes; and in the disease-courses ontology, morphology codes and behaviour codes were integrated, whereas a number of the data-validation rules refer to separate behaviour codes. More importantly, both ontologies contained many more classes and logical axioms than were required by the data-validation ontology (over 20,000 compared to some 4000 classes; and over 50,000/150,000 as opposed to some 6000 logical axioms), and would have impacted unfavourably on automatic-reasoning performance. Thus, the decision was taken to develop a dedicated ontology for the purpose of this work, particularly with a view to fulfilling the following main three requirements:

i) To provide the means of encapsulating the ENCR data-validation rules in a formal and unambiguous manner;
ii) To facilitate the integrity and maintenance of the rules by ensuring a unique, and uniquely addressable, repository of the rules;
iii) To utilise the automatic reasoning logic for the data validation and supplement it within a standalone computer programme only where the OWA was unable to make the necessary inferences.

The ontology was developed in the OWL sublanguage OWL DL in order to retain decidability to allow complete reasoning, as well as to take advantage of some critical underlying tools - such as the reasoning tools to infer logical consequences from a given set of asserted facts or axioms, and the Protégé ontology editor/user interface [25].

The ontology utilises the sixth edition of the TNM Classification of Malignant Tumours standard [26]; the third edition of the International Classification of Diseases for Oncology (ICD-O-3) [19]; the International Rules for Multiple Primary Cancers [27]; and ENCR recommendations (such as coding of basis of diagnosis) [28]. None of these standards have been formalised in ontologies and for the purpose of this work, the ICD-O-3 codes and the TNM edition 6 codes were recreated as separate ontologies to import into the main ontology.

### ENCR-JRC data–validation rules

The validation rules for the European CR core data set are described in [9]. For ease of interpretation, the rules are provided in a series of separate entity-relationship tables, which include:

i) unlikely and rare combinations of age and tumour type – an example of which is the combination of malignant extra-cranial and extra-gonadal germ cells (ICD-O-3 morphologies: 9060–9065, 9070–9072, 9080–9085, 9100–9105) with any of the ICD-O-3 topographies: C00-C55, C57-C61, C63-C69, C73-C750, C754-C768, C80; and age at diagnosis greater than 7;
ii) unlikely sex and topography combinations;
iii) valid combinations for basis-of-diagnosis and morphology and topography, such as basis of diagnosis specified as clinical investigation and ICD-O-3 morphology 9380 and ICD-O-3 topography C717;
iv) valid combinations for morphology and grade, such as ICD-O-3 morphologies: 9719, 9727, 9831, 9948 with grade 8 - NK cell (natural killer cell);
v) morphology codes and allowed topography codes, such as the combination of ICD-O-3 morphologies: 8160, 8161 with ICD-O-3 topographies C221, C239, and C240.

The validation rules also describe checks for permissible combinations of extent-of-disease and behaviour and TNM as well as specific checks for survival analysis and checks forinconsistencies of multiple primary malignant tumours, which if not identified can skew the statistics for incidence.

**Fig. 1:**
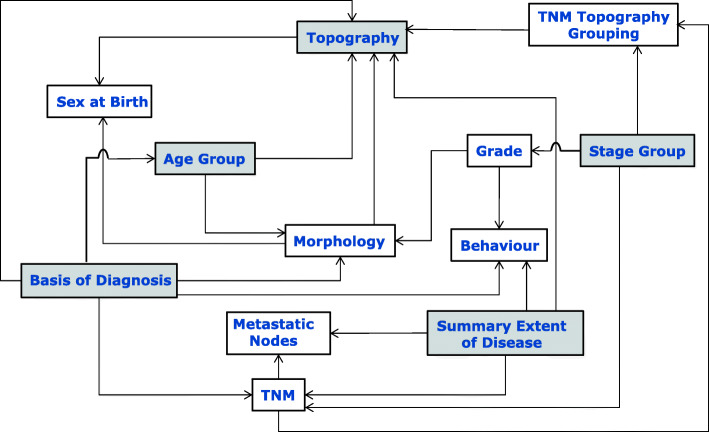
Representation of rule-dependencies between entities described by the ENCR entity-relationship data-validation tables. Lines depict dependencies (arrows point in the direction of dependency of one entity upon another entity). The shaded boxes depict direct and indirect dependencies on common entities as discussed in the text

Whereas presentation of the rules in such a way makes it easier to understand the relationships between a subset of specific entities, transcribing them directly to a semantic data model introduces a degree of inter-coupling between many of the associated entities. Figure 1 illustrates the entities^2^ (boxes) comprising the rule tables of [9] and their rule dependencies.

The degree of inter-coupling can be discerned to some extent by the number of relationships between the entities and also by the number of direct and indirect dependencies on common entities. In Fig. 1 for example, “Basis of Diagnosis” has a validation-rule dependence on “Topography”. It also has a dependence on “Morphology” that itself has a dependence on “Topography”. “Basis of Diagnosis” has a further indirect dependence on Topography via “TNM” and “TNM Topography Grouping”. Likewise, “Stage Group” has a direct dependence on “TNM Topography Grouping” as well as a further dependence via “TNM”. The entities involved in these types of dependencies are displayed as shaded boxes. Such dependencies tend to complicate the task of modelling entity-relationships in software and generally result in higher maintenance overheads.

**Fig. 2:**
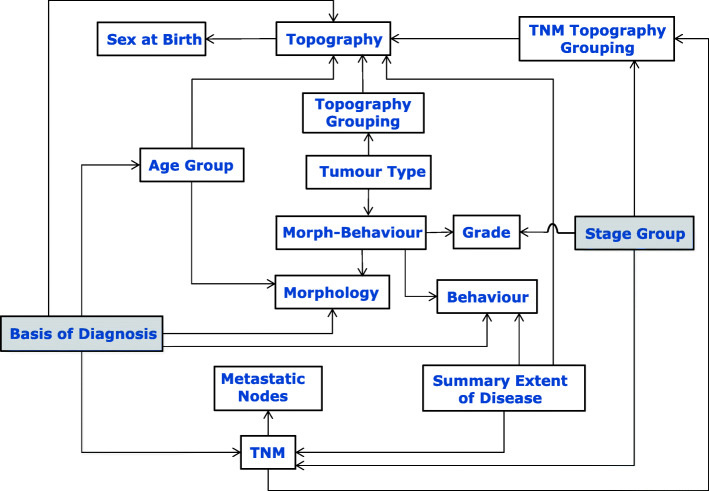
Introducing some appropriate extra entities can help reduce rule-dependencies between entities

Coupling can be reduced by refactoring some of the dependencies after adding a number of extra data entities. Figure 2 illustrates the situation after adding the extra data entities: “Topography Grouping” that groups topographies for different type of tumours; “Morph-Behaviour” that classifies the possible permutations of morphology and behaviour; and “Tumour Type” that classifies the possible tumour types on the basis of topography groupings and morphology-behaviour. These extra data entities were modelled on SEER’s histology/behaviour description categorisation [29], which is itself based on ICD-O-3.

**Fig. 3:**
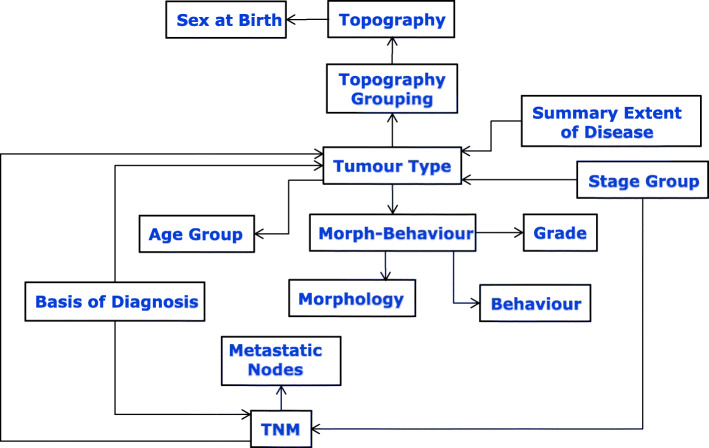
Further decoupling of dependencies resulting from alignment of TNM topography grouping and redefinition of basis-of-diagnosis in terms of tumour-type

The model however still suffers some drawbacks. In particular, the topography and morphology entities are not as decoupled as they could be – the reason being that the current rules for basis-of-diagnosis are described granularly in specific terms of morphology/morphology-topography, and age. Also the TNM topography groupings do not currently map in all cases to the topography-grouping definitions that reference to the tumour-type definitions (c.f. Fig. 2); for example, the TNM Topography Grouping (TNM 6th edition) entity for larynx includes the ICD-O-3 topography codes: C320, C321, C322, and C101 whereas the Topography Grouping entity includes codes: C320, C321, C322, C323, C328, and C329. Were these definitions to be redefined, then a cleaner model such as that shown in Fig. 3 could be realised.

### Transcribing the rules into the OWL ontology

The OWL ontology was developed on the entity-dependency model described in Fig. 2. The entities shown in the figure formed the main OWL classes and the rules were derived from the entity-relationship tables provided in [9].

The ICD-O-3 and the International Union Against Cancer (UICC) TNM tumour classifications do not currently exist in OWL format. Whereas others have developed OWL ontologies to address this need [13, 30], they are not comprehensive and were created with the specific aims of the respective studies in mind. A strength of OWL – which can nevertheless lead to a potential drawback – is that ontologies can be created in a number of ways, allowing an ontology to be tailored to a specific design need. An ontology tailored to one design constraint is not necessarily easily adaptable. In particular, the ontologies closest to the work presented here are described in [30]; however the ICD-O-3 ontology contained relatively few morphology classes and the TNM ontology (TNM edition 6) sub-classed the topographies under the stage groups. Our study required the full complement of the ICD-O-3 morphologies and for the TNM ontology it was preferable to sub-class the stage groups under the TNM topography groupings (since the former are generally dependent on the latter). It was therefore necessary to recreate separate ontologies for the OWL classes that mapped to ICD-O-3 and TNM. For the latter, edition 6 was used but the other editions (7 and 8) could be developed on the same basis.

The ability to import other ontologies from within a given ontology is nevertheless an important feature of OWL and will allow much faster development times as and when ontologies of standards suitable to cancer registration become available.

The classes and relationships were created using the Protégé tool. The expressivity of the description logic used in the ontology equated to SHIQ(D), which is less than OWL-DL’s full expressivity of SHOIN (D), but the validation tests did not require the functionality either of nominals (O) or of cardinality restrictions (N).

OWL provides a number of ways whereby rules can be encoded in an ontology. The rules however need to distinguish between what the JRC-ENCR validation process considers errors and warnings. The three following scenarios are used for handling CR data-coding violations: (i) direct violation of a strict rule resulting in a CR coding error via an OWL disjoint statement; (ii) “soft” violation of a rule via an unlikely condition that prompts a warning via an OWL restriction statement; and (iii) the conjunction of a number of conditions that together do not allow the code allocated by the cancer registry via OWL’s *class-subsumption* mechanism.

Scenario (i) can be handled simply by specifying certain classes disjoint from each other. For example the requirement that in situ behaviour (code 2) must have basis-of-diagnosis given as ether via cytology (code 5) or histology of primary tumour (code 7) can be encoded by the statement using description-logic syntax:

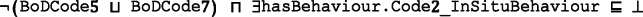



This statement essentially states that any basis-of-diagnosis, other than code 5 or code 7, which is associated with a behaviour of type “in situ” (code 2) is a member of the empty set.

Scenario (ii) can be handled via sub-classing. The class with a “soft” condition can be made a sub-class of a restriction. Thus, to model the fact that hepatoblastoma is unlikely to occur above the age of 5, its associated class can be made a sub-class of the restriction:

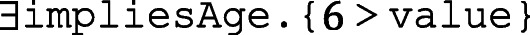



Scenario (iii) can be handled using defined classes. Defined classes are those that describe the necessary and sufficient conditions for any other class to be subsumed by them. It is the means by which a closed-world set of conditions can be expressly stated in OWL. For example, the definition for a basis-of-diagnosis described by the code “Clinical” can be specified by the defined class:

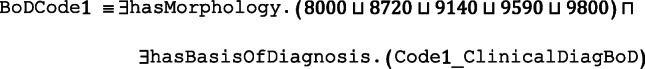



which means that if the parameters specified by an individual CR case record include a clinical basis-of-diagnosis (Code1_ClinicalDiagBoD) and morphology either of 8000, 8720, 9140, 9590, or 9800 and a behaviour not given by “in situ” (c.f. axiom above for basis-of-diagnosis codes and behaviour code: Code2_InSituBehaviour) [28], then the OWL reasoner will be able to subsume the record under the class BoDCode1 and thereby validate the basis-of-diagnosis field of the record. Conversely if the morphology field of the record is outside these prescribed values, or a behaviour of in situ (code 2) is specified, the reasoner will not be able to subsume the record under the BoDCode1 class and a mismatch in the basis-of-diagnosis code can be inferred.

## Results

**Fig. 4:**
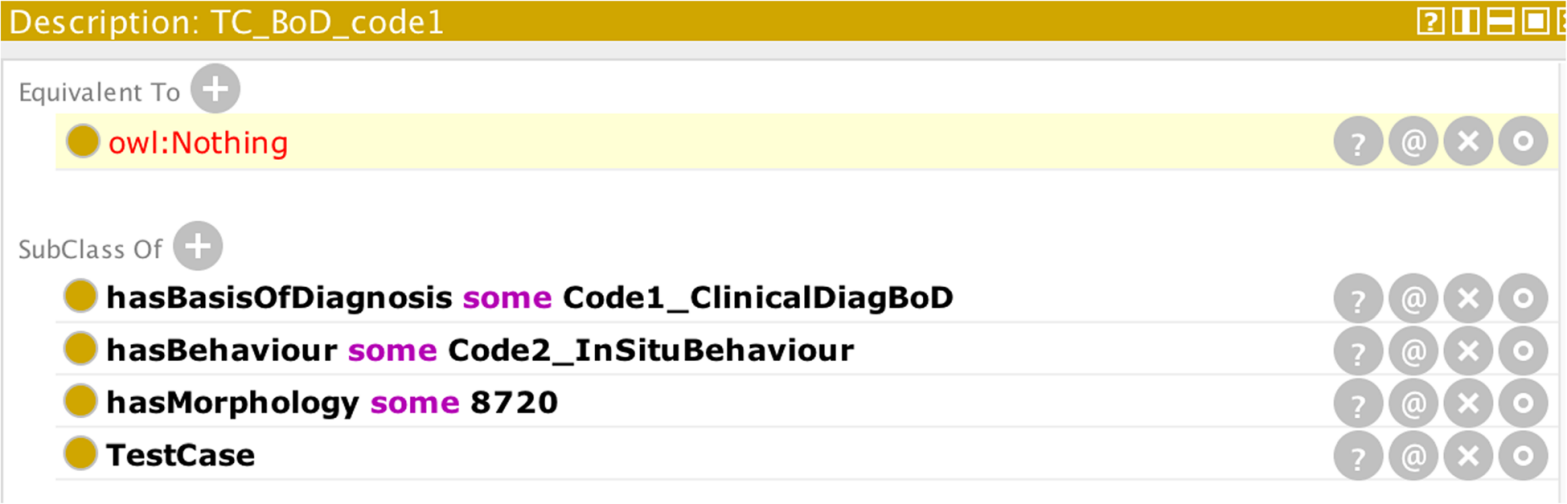
Reasoner output (highlighted in yellow) on the basis of the given input parameters (illustrating the hard-restraint scenario)

To demonstrate how the ontology works in practice, Fig. 4 shows the result in Protégé when the OWL reasoner (FaCT++) is run on the input case parameters: morphology = 8720, basis-of-diagnosis code = Code1_ClinicalDiagBoD (diagnosis made before death but without reference to any of the other bases of diagnosis), and behaviour = Code2_InSituBehaviour (tumour confined to primary site).

**Fig. 5:**
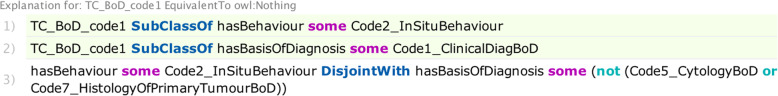
Specific explanation of why the reasoner returned an empty set in Fig. 4. The highlighted statements (1 and 2) refer to the axioms asserted in the input case record. Statement 3) is the reason behind the empty set

**Fig. 6:**
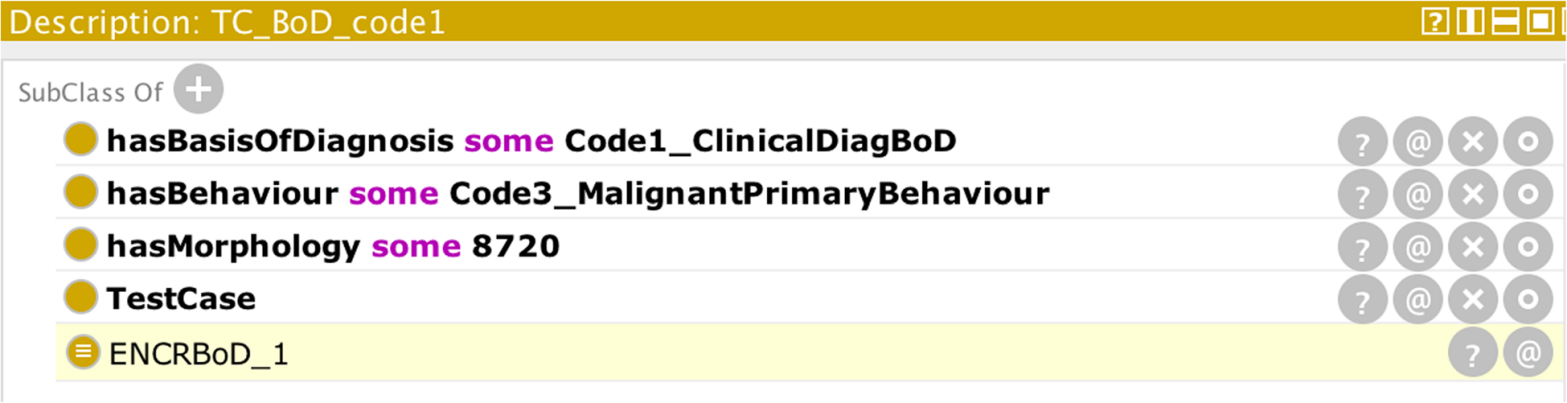
Output from reasoner (highlighted in yellow) after correction of behaviour code

The reasoner has determined that the input class is an empty set. Further querying of the reasoner (Fig. 5) reveals that the basis-of-diagnosis code 1 (ENCRBoD_1) is disjoint with in situ behaviour as discussed earlier:

Correcting the value for behaviour and re-running the reasoner now yields (Fig. 6):

where basis-of-diagnosis has been ascertained as correct and the input class fulfils the necessary and sufficient conditions for it to be subsumed under the ENCRBoD_1 class.

**Fig. 7:**
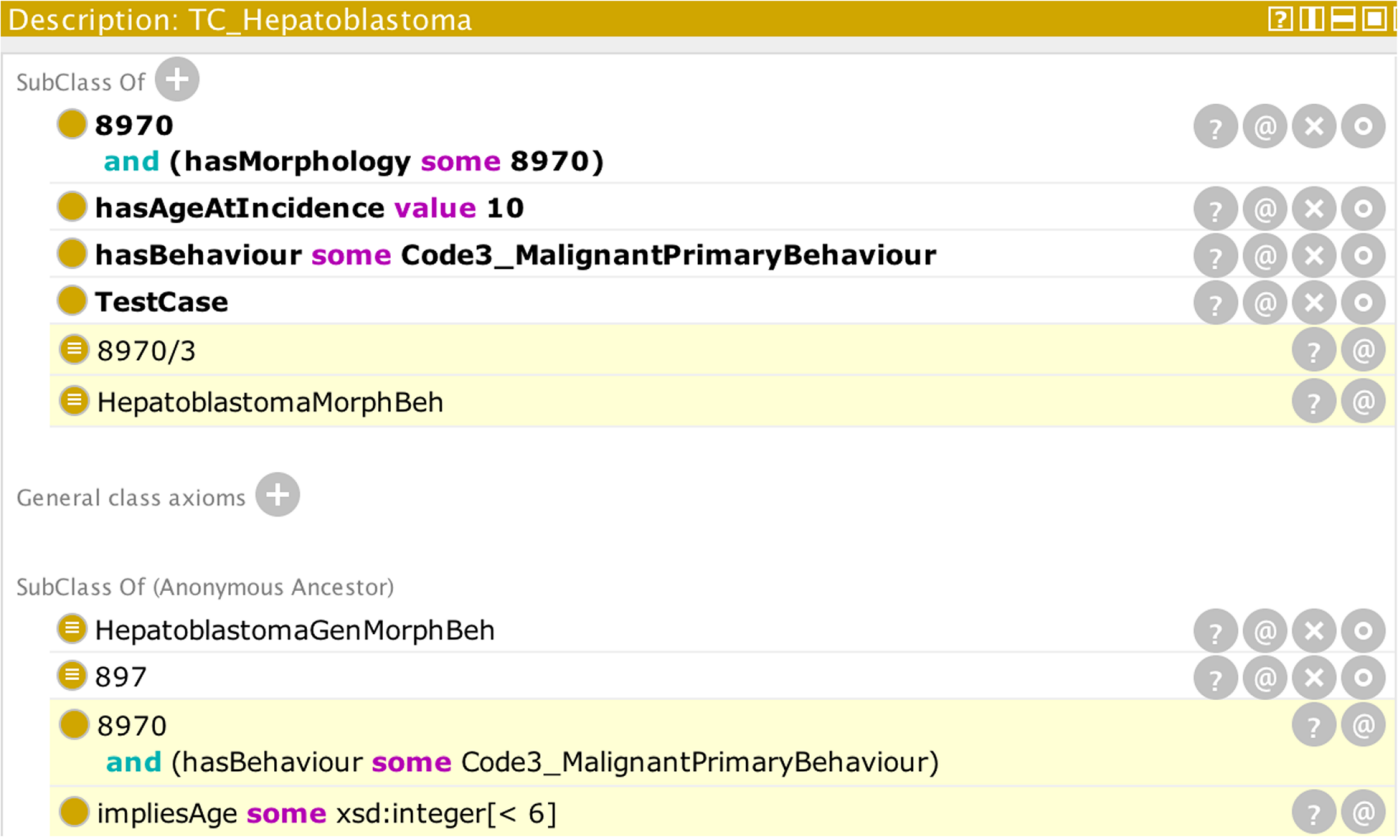
Output from the reasoner (highlighted in yellow) on the basis of the given input parameters (illustrating the “soft-error” scenario)

Figure 7 illustrates the soft constraint discussed in scenario (ii). The reasoner has correctly determined that an ICD-O-3 morphology code of 8970 and behaviour of malignant primary site has morphology-behaviour of hepatoblastoma.

Even though a value of ten has been input for “age of incidence”, the reasoner has not returned an empty set as for a “hard error”. Whereas the condition could have been encoded with a disjoint axiom, there are potentially some rarer types of cases for which such a combination of parameters is valid. Before determining the validity of the case, it is necessary also to check all the classes from which the set of parameters are sub-classed. In Fig. 7 for example, the last line shows that the combination of some of the input parameters infer an age less than six and this is in contradiction to the input age of ten.

**Fig. 8:**
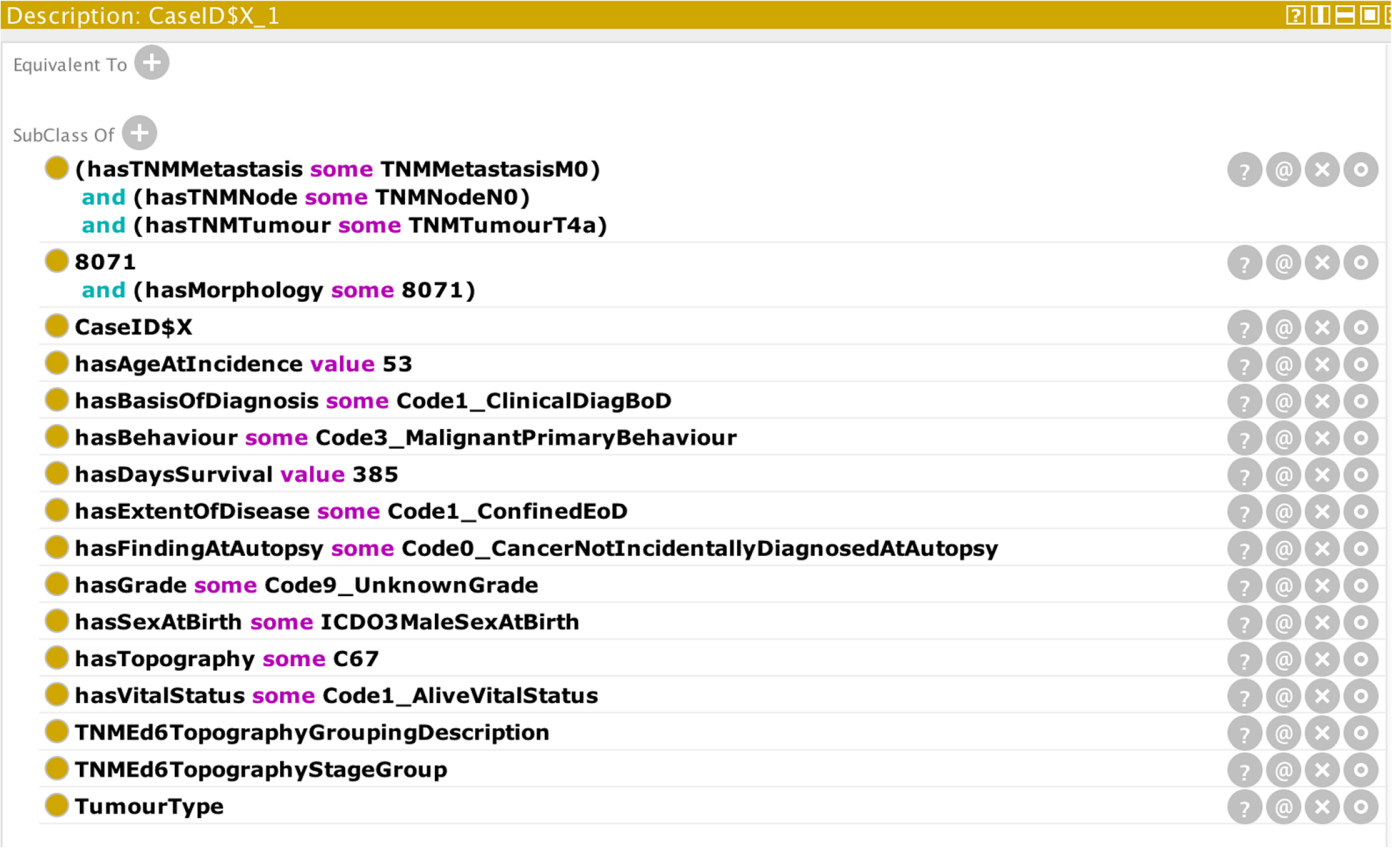
Input parameters of a comprehensively described cancer case

The previous examples addressed just one or two cancer-case codes. A cancer case consists of a number of core and optional codes. Figure 8 illustrates an OWL class (called CaseID$X_1) for a comprehensively described cancer case relating to a fictitious male patient of 53 years of age at diagnosis.

**Fig. 9:**
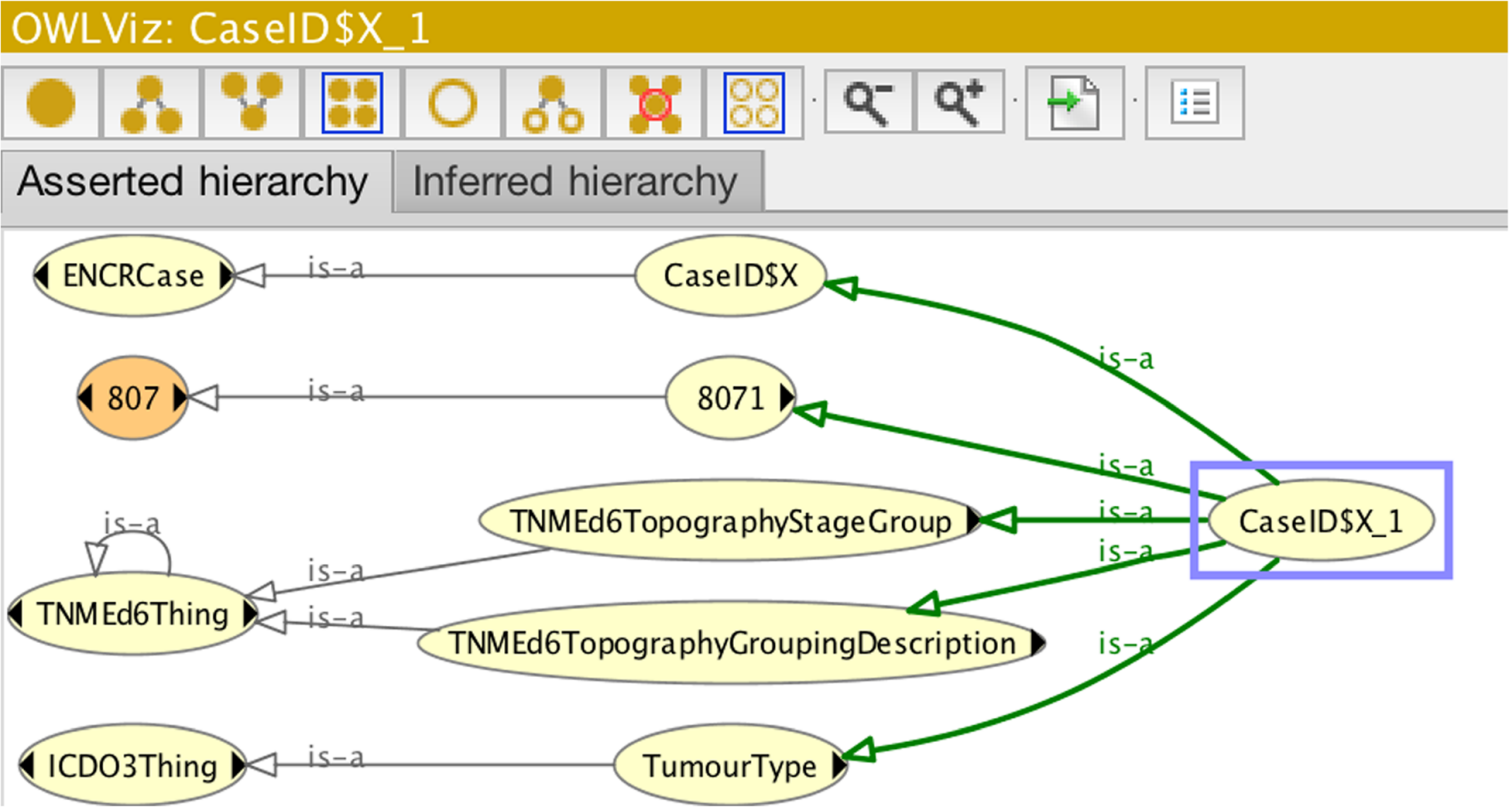
Asserted OWL-class hierarchy of the OWL class CaseID$X_1. Lighter-coloured nodes depict primitive classes, while darker-coloured nodes depict defined classes (e.g. the three-digit morphology class: 807)

Figure 9 shows in diagrammatic form the asserted class hierarchy of the OWL class CaseID$X_1 illustrated in Fig. 8. It can be seen from Fig. 8 that the class CaseID$X_1 is sub-classed from five named parent classes (more clearly illustrated in Fig. 9) and a number of unnamed parent classes.

Of the named parent OWL classes, the class “8071” describes the specific morphology class, which together with the specified restriction on behaviour will allow an automatic determination of “Morph-Behaviour” data entity (c.f. Fig. 2). The class “TumourType” equates to the “Tumour Type” data entity of Fig. 2, allowing the reasoner to determine if there is a permitted tumour-type classification for the given ICD-O-3 codes for morphology, behaviour, and topography (c.f. Fig. 8). The classes “TNMEd6TopographyStageGroup” and “TNMEd6TopographyGroupingDescription” equate to the respective data entities “Stage Group” and “TNM Topography Grouping”. Together they allow an automatic determination of the specific TNM stage group on the basis of the restrictions on TNM codes and topography. The class “CaseID$X” allows the reasoner to determine if the specific cancer-case class CaseIDX_1 contains all the mandatory fields or not.

**Fig. 10:**
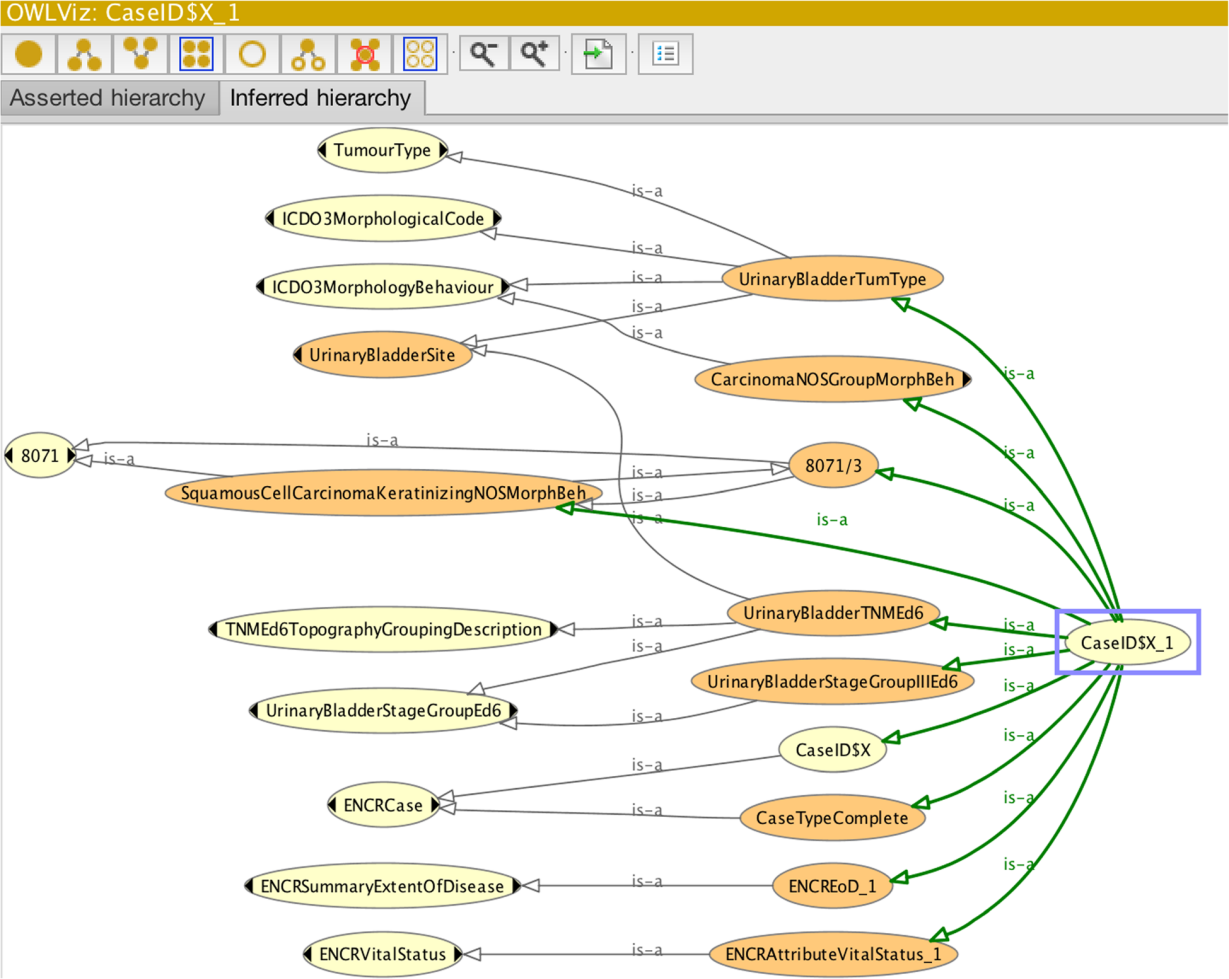
Results from the reasoner of class CaseID$X_1 specified in Fig. 8. Lighter-coloured nodes depict primitive classes, and darker-coloured nodes depict defined classes

Figure 10 shows the reclassification of the parent classes inferred by the reasoner on the basis of the underlying rules previously encoded in the ontology.

Bearing in mind the input parameters in Fig. 8, it may be observed that the reasoner has:

(i) determined that the morphology, behaviour, and topography combination constitute a pre-classified tumour-type (urinary bladder);
(ii) determined a squamous cell carcinoma (keratinising) morphology-behaviour and further determined that it belongs to a group of carcinoma morphology-behaviours (“CarcinomaNOSGroupMorphBeh”) – which carries an implication for age greater than five, though this fact is not visible in the diagram;
(iii) confirmed the validity of the assigned vital-status code;
(iv) confirmed that the summary extent-of-disease code is valid;
(v) determined a TNM stage group III for the TNM topography grouping “Urinary Bladder”;
(vi) confirmed that case class contains all the mandatory fields (“CaseTypeComplete”);

It is noted however that the reasoner has not explicitly confirmed the basis-of-diagnosis code. The reasoner’s “silence” on the basis-of-diagnosis value corresponds to the error-type (iii) scenario. The reasoner was unable to find a class that subsumed the ascribed basis-of-diagnosis code. Indeed the ENCR rules for basis of diagnosis only permit a restricted range of morphologies for code 1 (clinical basis-of-diagnosis). Using the Protégé interface therefore requires users to verify that all the codes have been confirmed before ascertaining that the case record is correct.

**Fig. 11:**
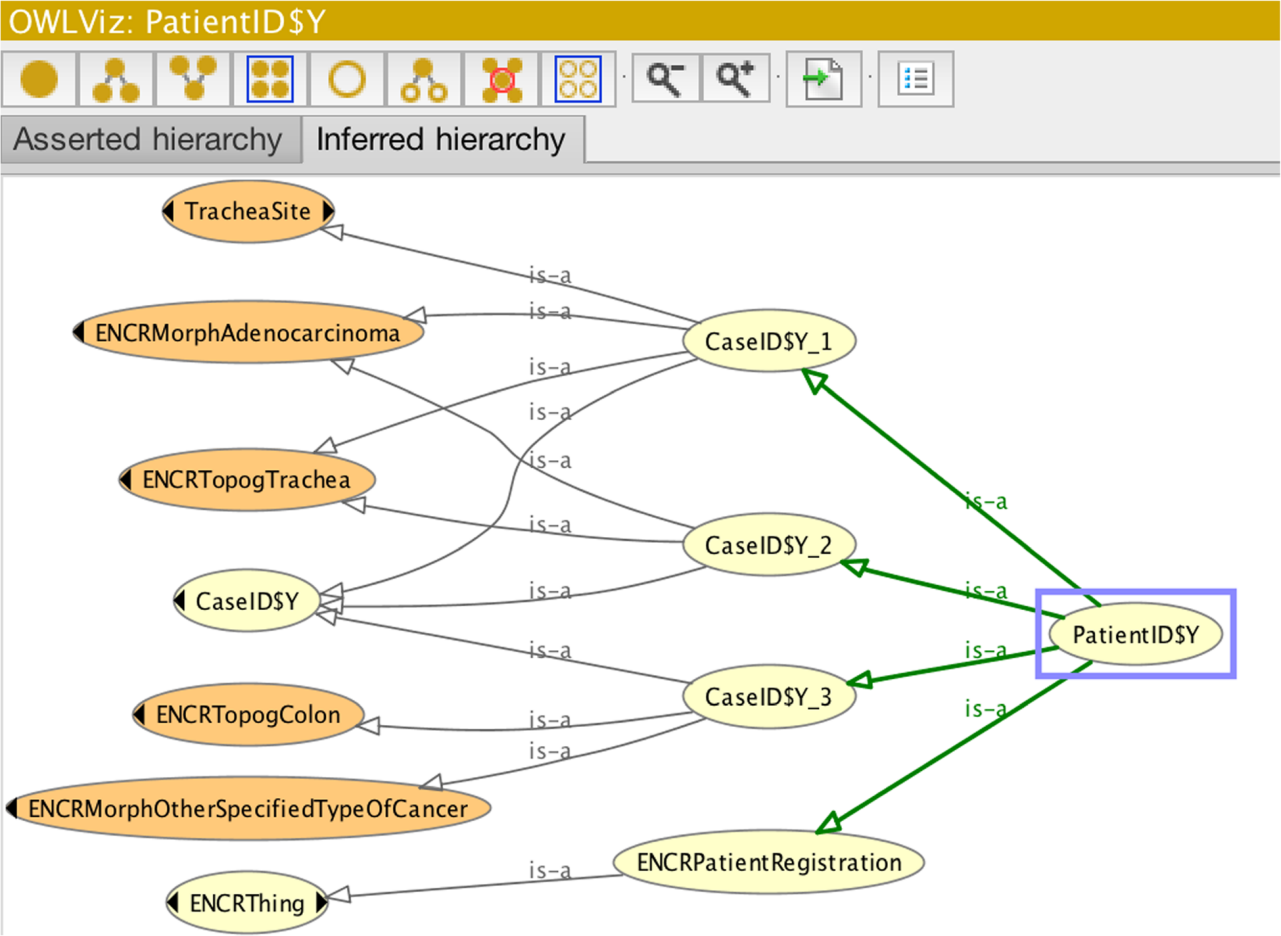
Class hierarchy inferred by the reasoner of three cancer cases of a single patient, in which an instance of a multiple-primary tumour is evident (CaseID$Y_1 and CaseID$Y_2). Lighter-coloured nodes depict primitive classes, and darker-coloured nodes depict defined classes

The ontology also allows individual cases pertaining to a single cancer patient to be considered together – this is important for determining conditions relating to multiple-primary tumours. The rules for multiple-primary tumours are relatively complex [27] and are best handled via a programming interface; however they can also be visualised in Protégé and interpreted with knowledge of the rules. An example is taken for an imaginary patient (PatientID$Y) with three separate cancer-case registrations. In order to keep the visualisation as clear as possible, only the morphology, topography, and behaviour fields are used (since these are all that are required by the rules). The other field variables can be validated in the manner discussed in the previous examples. Case 1 is defined by ICD-O-3 topography code C33 and morphology code 8550; case 2, by topography code C34 and morphology code 8140; and case 3, by topography code C18 and morphology code 8936. All three cases have default behaviour Code3_MalignantPrimaryBehaviour. Cases 1 and 2 both have their topography codes and their morphology codes in single topography and morphology groups and thus should only be considered as a single case. Case 3 in contrast has a topography code in a different group to those corresponding to cases 1 and 2 and is therefore indeed to be considered as an independent case. Figure 11 illustrates Protégé’s rendering of the inferred class hierarchy of the three cases, from which it can be seen that both case 1 (CaseID$Y_1) and case 2 (CaseID$Y_2) are subsumed by the same parent classes for topography group (ENCRTopogTrachea) and morphology group (ENCRMorphAdenomacarcinoma), whereas case 3 (CaseID$Y_3) is subsumed by a different topography and morphology group.

### Limitations of the Protégé visualisation tool and the need for a dedicated programme interface

Whereas the Protégé tool is extremely useful for visualising the inferred class dependencies, it suffers a number of limitations with regard to alerting users to some types of errors as indicated above, and particularly regarding the more complex types of checks such as those for multiple-primary tumours. Moreover, CR core data files consist of hundreds of thousands or even millions of case records and it would be impractical to verify the records individually.

A dedicated programing interface could overcome these limitations and allow clearer explanations to be provided in the associated output logs – for example, it would be possible to handle each of the three error-trapping scenarios discussed earlier and to log them appropriately as errors or warnings, with more specific information regarding the respective issue. The addition of conditional statements within the programme can also increase the expressivity of the logic where absolutely required, but has the drawback of increased code maintenance overheads.

OWL provides an application-programming interface (OWL-API, [31]) to support the development of software for automating the data-validation process that in turn would enable batch processing of CR data files. Additional control could also be given to the more complex types of tests requiring checks over permutations of cancer cases (as for example in the checks for multiple-primary tumours).

One particularly useful aspect of OWL-API lies in its ability to poll any given class in both its asserted and inferred hierarchies, potentially providing the means in some instances to perform automatic error correction.

The next step will be to develop this software as open source once the final semantic data model has been agreed and the associated ontologies updated. Prototype software was developed in the course of the work to ascertain the viability in terms of performance. Validation of 100,000 records took approximately 10 min on an Intel Celeron low-end processor, without any optimisation of code. The process read a batch of records (cancer cases) from the CR data file, constructing each record as a separate OWL class and adding them to the ontology. Thereafter the reasoner (Hermit, for ease of portability across hardware platforms) was invoked from within the programme and the resulting inferences checked for completeness or error for each class. Errors were written to an error log. After terminating the reasoner, the cancer-case classes were removed and the next batch of records was read in until the whole file had been ingested. Batch reading of records avoided bloating the ontology and unnecessarily affecting performance. Real-time results of the validation process for an entire cancer-registry data set is however not a fundamental requirement and the checks can always be run in background mode.

There is also the need for a pre-processing stage to capture basic data errors, such as typographic errors and missing mandatory variables that can be trapped more efficiently outside the ontology. In the current data-cleaning process this step is performed automatically via a dedicated software programme during the data upload procedure where the formats of the core data-files are checked against the file format specified in the data-call protocol. Data files have to pass this initial check before they can be uploaded for further processing. As a future step that will serve to complement the formalisation of the inter-variable checks in OWL, the core data template will be translated into an RDF data-constraint language, such as ShEx [32] or SHACL [33].

## Discussion

The foregoing examples have illustrated the power and usefulness of the ontology-based approach towards CR data validation, even just using the readily available Protégé OWL user interface as illustrated in the preceding examples. The ontology could find immediate application in local cancer registries to visualise the dependencies between the different data entities. The inferences made by any of the reasoning tools, bundled with Protégé, provide the reasons for which incorrect data-field combinations that have been determined.

Initiatives are currently underway to allow greater access to aggregated CR data sets following a more federated data approach. The further development of the CR-data ontology beyond the core-data variables could lead to the availability of richer aggregated data sets that could be used for inter-regional studies. It would be important however to align further development with the initiatives addressing other aspects of cancer-registry work discussed under the Implementation section.

Consideration of the wider applicability of ontologies is particularly important to encourage reuse and avoid the creation of many separate ontologies addressed to specific applications, leading to an eventual unmanageable set of unlinked semantic resources. This is critical if not just from the perspective of ensuring a common, harmonised, and consistent and set of metadata. In this regard, the applicability and versatility of OWL ontologies can have detrimental consequences. Even within a specialist field, it is apparent that data serves many different purposes. If ontologies are developed with a sole purpose in mind there is the danger that practitioners in the field will have to contend with a number of specific ontologies for the different data purposes, and worse still, a number of different naming conventions for the same entities. Ontologies should therefore be designed circumspectly with a view to possible other applications within a specific domain. Herein lies a major challenge; reuse of ontologies is not as straightforward as it may first appear [34, 35]. Ontologies are models of reality in some given domain and these models can be subjective even for experts within the same domain and may have further dependences on the specific types of tasks addressed within the domain. Moreover, performance issues in terms of automatic reasoning may place strict constraints on the ontology design itself. In particular, reuse of medical ontologies is challenging due to their size and can entail significant costs outweighing those related to a new implementation [35]. As an additional complication, medicine is a dynamic domain potentially requiring updates to standard ontologies in relatively short time-frames thereby imposing integrity issues on any dependent ontologies.

Further investigation is therefore required to understand how best to develop a single ontology that is able to address the different tasks within a cancer registry. Once stable, the ontology developed in this work will be used to validate the whole set of ENCR data, at which stage it could possibly be proposed as the standard ENCR data-validation tool. It would thereby benefit from regular maintenance and continual improvements on the basis of recommendations of CRs that may help it position itself as an eventual contender for such a unified ontology.

## Conclusions

It has been shown how the implementation of ontology-based data-validation tools can benefit the processes underlying the compilation of European population-based cancer indicators. The benefits can be appreciated from the following considerations:

Firstly, ontologies provide the means of expressing the data-validation rules in a formal sense, thereby removing ambiguities and the potential for consequent misinterpretation, as well as helping to identify unnecessary data-coupling relationships. A formal representation of the rules avoids potential ambiguities.

Secondly, data-validation tools can be derived naturally from the ontology and if the system is designed with due care, any changes to the ontology will automatically be reflected in the data-validation tool, thereby resolving synchronisation and versioning issues between the updated rule base and the associated validation software.

Thirdly, the names of the classes specified in the ontology are directly associated with the value-domain specifications of the underlying metadata. Owing to the fact that in OWL all classes are defined by Internationalised Resource Identifiers (IRIs), they could in principle be used directly as unique meta-data component identifiers, thereby removing the need to map local data to a number of different data-reporting/submission formats.

Fourthly, carefully crafted ontologies offer a scalable solution for further development. Ontologies can import other ontologies, thereby reducing development time and maintenance effort, and allowing data modellers to focus more on the specific domain of interest whilst reutilising the work of others. Furthermore, new axioms can be added to the ontology to reflect dynamically changing processes or to allow piece-wise extensions without necessarily breaking any previous underlying dependencies.

Fifthly, ontologies can help federate data sources by providing a harmonised and transparent tool for validating data to an appropriate standard at the local level, thereby enabling provision of locally aggregated data and avoiding the need for duplicating the single-record data at a centralised level with all the attendant problems of data maintainability, data integrity, and data-privacy/protection issues. This aspect in relation to ensuring data conform to the FAIR (Findable, Accessible, Interoperable, and Re-usable) guiding principles [36] becomes particularly interesting when integrated into an appropriate higher-level framework based on semantic interoperability and linked open data [37, 38].

Finally, ontologies lend themselves to description logic frameworks and all the advantages they bring (such as reasoning algorithms and potential error correction) based on many years of research.

With respect to CR data, an ontology-based data validation tool opens up the possibility towards decentralising the data-validation process at supranational level by ensuring an unequivocal set of data rules that could be applied in a harmonised way at the local level. Moreover, with little extra maintenance of the underlying data-validation software, the ontology can be extended/updated with new sets of data axioms as and when necessary.

## Availability and requirements

Project name: ENCR core-data ontology

Project home page: https://data.jrc.ec.europa.eu/dataset/efd6acd1-2cfd-401b-b5ea-d05f8efbb123

Operating system(s): Platform independent

Programming language: Web Ontology Language (OWL)

Other requirements: Ontology editor (e.g. Protégé Desktop v.5.5.0)

License: BSD 2-clause licence (Protégé)

No restrictions on use by non-academics

## Data Availability

The datasets generated during the current study are available in the Joint Research Centre data catalogue repository, https://data.jrc.ec.europa.eu/dataset/efd6acd1-2cfd-401b-b5ea-d05f8efbb123.

## Abbreviations

API: Application-programming interface
CR: Cancer registry
CWA: Closed world assumption
DL: Description logic
ECIS: European cancer information system
ENCR: European network of cancer registries
FAIR: Findable, accessible interoperable, re-usable
IARC: International agency for research on cancer
GDPR: General data protection regulation
ICD-10: International classification of diseases 10th revision
ICD-O-3: International classification of diseases for oncology 3rd edition
IRI: International resource identifier
JRC: Joint research centre
NCIt: North American national cancer institute thesaurus
OWA: Open world assumption
OWL: Web ontology language
OWL-API: Web ontology language application-programming interface
RDF: Resource description framework
SHACL: Shapes constraint language
ShEx: Shape expressions – languages for validating and describing RDF
SHIQ(D), SHOIN(D): Expressivities of description logic
SNOMED CT: Systematized nomenclature of medicine – clinical terms
SPARQL: SPARQL protocol and RDF query language
TBox: Terminological box/component
TNM: TNM classification of malignant tumours (T: size of original tumour; N: Involvement of regional lymph nodes; M: Presence of distant metastasis)
UICC: International union against cancer
URI: Uniform resource identifier

## Footnotes

1 EUROpean CAncer REgistry based study on survival and care of cancer patients.

2 To clarify a point of possible confusion – the entity “Metastatic Nodes” is a separate, independent entity from the value of M within the entity TNM. The number of metastatic nodes is an additional variable to the values of TNM in the core data set.
